# Author Correction: Endothelial cell heterogeneity and microglia regulons revealed by a pig cell landscape at single-cell level

**DOI:** 10.1038/s41467-022-34498-w

**Published:** 2022-11-08

**Authors:** Fei Wang, Peiwen Ding, Xue Liang, Xiangning Ding, Camilla Blunk Brandt, Evelina Sjöstedt, Jiacheng Zhu, Saga Bolund, Lijing Zhang, Laura P. M. H. de Rooij, Lihua Luo, Yanan Wei, Wandong Zhao, Zhiyuan Lv, János Haskó, Runchu Li, Qiuyu Qin, Yi Jia, Wendi Wu, Yuting Yuan, Mingyi Pu, Haoyu Wang, Aiping Wu, Lin Xie, Ping Liu, Fang Chen, Jacqueline Herold, Joanna Kalucka, Max Karlsson, Xiuqing Zhang, Rikke Bek Helmig, Linn Fagerberg, Cecilia Lindskog, Fredrik Pontén, Mathias Uhlen, Lars Bolund, Niels Jessen, Hui Jiang, Xun Xu, Huanming Yang, Peter Carmeliet, Jan Mulder, Dongsheng Chen, Lin Lin, Yonglun Luo

**Affiliations:** 1 Lars Bolund Institute of Regenerative Medicine, Qingdao-Europe Advanced Institute for Life Sciences, BGI-Qingdao, BGI-Shenzhen, Qingdao, China; 2 Department of Biomedicine, Aarhus University, Aarhus, Denmark; 3 BGI-Shenzhen, Shenzhen, China; 4 College of Life Sciences, University of Chinese Academy of Sciences, Beijing, China; 5 Department of Biology, University of Copenhagen, Copenhagen, Denmark; 6 Steno Diabetes Center Aarhus, Aarhus University Hospital, Aarhus, Denmark; 7 Department of Neuroscience, Karolinska Institutet, Stockholm, Sweden; 8 MGI, BGI-Shenzhen, Shenzhen, China; 9 Laboratory of Angiogenesis and Vascular Metabolism, Center for Cancer Biology, VIB, Leuven, Belgium; 10 Department of Oncology, Leuven Cancer Institute, KU Leuven, Leuven, Belgium; 11 College of Basic Medicine, Qingdao University, Qingdao, China; 12 School of Basic Medical Sciences, Binzhou Medical University, Yantai, China; 13 Institute of Systems Medicine, Chinese Academy of Medical Sciences, Peking Union Medical College, Beijing, China; 14 Suzhou Institute of Systems Medicine, Suzhou, China; 15 Aarhus University of Advanced Studies (AIAS), Aarhus University, Aarhus, Denmark; 16 Department of Protein Science, Science for Life Laboratory, KTH-Royal Institute of Technology, Stockholm, Sweden; 17 Department of Obstetrics and Gynecology, Aarhus University Hospital, Aarhus, Denmark; 18 Department of Immunology, Genetics and Pathology, Uppsala University, Uppsala, Sweden; 19 IBMC-BGI Center, the Cancer Hospital of the University of Chinese Academy of Sciences (Zhejiang Cancer Hospital), Institute of Basic Medicine and Cancer (IBMC), Chinese Academy of Sciences, Hangzhou, China; 20 Center for Biotechnology, Khalifa University of Science and Technology, Abu Dhabi, United Arab Emirates

**Keywords:** RNA sequencing, Systems analysis, Databases, Microglia, Angiogenesis

Correction to: *Nature Communications* 10.1038/s41467-022-31388-z, published online 24 June 2022

The original version of this Article contained an error in the <Endothelial cell heterogeneity> section of the Results, which incorrectly read ‘<Our results showed that EndMT cells can be clearly identified in adipose tissues with co-expression of PECAM1 and ACTA2 (Fig. 3i), as well as VWF and TAGLN, in a small fraction of ECs (Fig. 3j).>.’ The correct version states ‘<Our results showed that EndMT cells can be clearly identified in adipose tissues with co-expression of VWF and TAGLN (Fig. 3i), as well as PECAM1 and ACTA2 (Fig. 3j), in a small fraction of ECs.>’.

The original version of this Article contained an error in the legend to Figure 3, which incorrectly read ‘<i. Representative Immunofluorescence staining images of PECAM1 and ACTA2 in adipose tissue. Arrow (red) indicates ECs expressing both PECAM1 and ACTA2. Arrow (green) indicates ECs only expressing PECAM1 (*n* = 3). j. Representative Immunofluorescence staining images of VWF and TAGLN in adipose tissues. Arrow (red) indicates ECs expressing both VWF and TAGLN. Arrow (green) indicates ECs only expressing VWF (*n* = 3). k. IHC of FABP4 in ECs and adipose tissues. Arrows (red) indicate ECs. Arrows (green) indicate adipocytes. Control was stained with an antibody against a gene not expressed in adipose tissues (*n* = 3).>.’ The correct version states ‘<i. Representative Immunofluorescence staining images of VWF and TAGLN in adipose tissues. Arrow (red) indicates ECs expressing both VWF and TAGLN. Arrow (green) indicates ECs only expressing VWF (*n* = 3). j. Representative Immunofluorescence staining images of PECAM1 and ACTA2 in adipose tissue. Arrow (red) indicates ECs expressing both PECAM1 and ACTA2. Arrow (green) indicates ECs only expressing PECAM1 (*n* = 3). k. IHC of FABP4 in ECs and adipose tissues. Arrows (green) indicate ECs. Arrows (red) indicate adipocytes. Control was stained with an antibody against a gene not expressed in adipose tissues (*n* = 3).>’.

The original version of this Article contained an error in the <Pre-processing and quality control of scRNA-seq and snRNA-seq data> section of the Methods, which incorrectly read ‘<Cells were only retained if the number of detected genes were greater than 200 and less than 5000 and the percentage of detected mitochondrial genes (percentage of MT) was less than 30%.>.’ The correct version states ‘<Cells were only retained if the number of detected genes were greater than 200 and less than 5000 and the percentage of detected mitochondrial transcripts from MT genes (ATP6, ATP8, COX1, COX2, COX3, CYTB, ND2, ND3, ND4, ND4L, ND5, ND6) was less than 30%. The Pig ND1 gene was not included in MT-based filtering due to high sequence variant in pigs.>’.

This has been corrected in both the PDF and HTML versions of the Article.

