# Supplementary material for: Endothelial cell heterogeneity and microglia regulons revealed by a pig cell landscape at single-cell level
Source: Nat Commun. 2022 Jun 24;13:3620. doi: 10.1038/s41467-022-31388-z (PMC9232580; doi:10.1038/s41467-022-31388-z)
Supplement: Supplementary file 1 — Supplementary Information [file 41467_2022_31388_MOESM1_ESM.pdf]

SUPPLEMENTARY INFORMATION FOR

Endothelial Cell Heterogeneity and Microglia Regulons Revealed by A Pig Cell Landscape at Single-cell Level

Fei Wang[1,2,3,†], Peiwen Ding[3,4†], Xue Liang[1,5, †], Xiangning Ding[3,4,†], Camilla Blunk Brandt [2,6,†], Evelina Sjöstedt[7], Jiacheng Zhu[3,4], Saga Bolund[7], Lijing Zhang[3,4,8], Laura P.M.H. de Rooij[9,10], Lihua Luo[3,4], Yanan Wei[3,11], Wandong Zhao[3,11], Zhiyuan Lv[3,11], János Haskó[2], Runchu Li[3,11], Qiuyu Qin[3,11], Yi Jia[3,11], Wendi Wu[3,11], Yuting Yuan[12], Mingyi Pu[3,11], Haoyu Wang[3,4], Aiping Wu[13, 14], Lin Xie[8], Ping Liu[8], Fang Chen[8], Jacqueline Herold[2], Joanna Kalucka[2,6,15], Max Karlsson[16], Xiuqing Zhang[3,4], Rikke Bek Helmig[17], Linn Fagerberg[16], Cecilia Lindskog[18], Fredrik Pontén[18], Mathias Uhlen[7,16], Lars Bolund[1,2], Niels Jessen[6], Hui Jiang[8], Xun Xu[3], Huanming Yang[3,19], Peter Carmeliet[2,9,10, 20], Jan Mulder[7], Dongsheng Chen[3, 13, 14,*], Lin Lin[2,6,*], Yonglun Luo[1,2,3,6,19,*]

1. Lars Bolund Institute of Regenerative Medicine, Qingdao-Europe Advanced Institute for Life Sciences, BGI-Qingdao, BGI-Shenzhen, Qingdao, China.
2. Department of Biomedicine, Aarhus University, Aarhus, Denmark.
3. BGI-Shenzhen, Shenzhen, China.
4. College of Life Sciences, University of Chinese Academy of Sciences, Beijing, China.
5. Department of Biology, University of Copenhagen, Copenhagen, Denmark.
6. Steno Diabetes Center Aarhus, Aarhus University Hospital, Aarhus, Denmark.
7. Department of Neuroscience, Karolinska Institutet, Stockholm, Sweden
8. MGI, BGI-Shenzhen, Shenzhen, China
9. Laboratory of Angiogenesis and Vascular Metabolism, Center for Cancer Biology, VIB, Leuven, Belgium
10. Department of Oncology, Leuven Cancer Institute, KU Leuven, Leuven, Belgium
11. College of Basic Medicine, Qingdao University, Qingdao, China.
12. School of Basic Medical Sciences, Binzhou Medical University, Yantai, China.
13. Institute of Systems Medicine, Chinese Academy of Medical Sciences, Peking Union Medical College, Beijing, China
14. Suzhou Institute of Systems Medicine, Suzhou, China
15. Aarhus University of Advanced Studies (AIAS), Aarhus University, Aarhus, Denmark
16. Department of Protein Science, Science for Life Laboratory, KTH-Royal Institute of Technology, Stockholm, Sweden
17. Department of Obstetrics and Gynecology, Aarhus University Hospital, Aarhus, Denmark
18. Department of Immunology, Genetics and Pathology, Uppsala University, Uppsala, Sweden.
19. IBMC-BGI Center, the Cancer Hospital of the University of Chinese Academy of Sciences (Zhejiang Cancer Hospital), Institute of Basic Medicine and Cancer (IBMC), Chinese Academy of Sciences, Hangzhou, China.
20. Center for Biotechnology, Khalifa University of Science and Technology, Abu Dhabi, United Arab Emirates.

† These authors contributed equally: Fei Wang, Peiwen Ding, Xue Liang, Xiangning Ding, Camilla Blunk Brandt.

* These authors jointly supervised this work:

Dongsheng Chen, cds@ism.pumc.edu.cn

Lin Lin, lin.lin@biomed.au.dk

Yonglun Luo, alun@biomed.au.dk

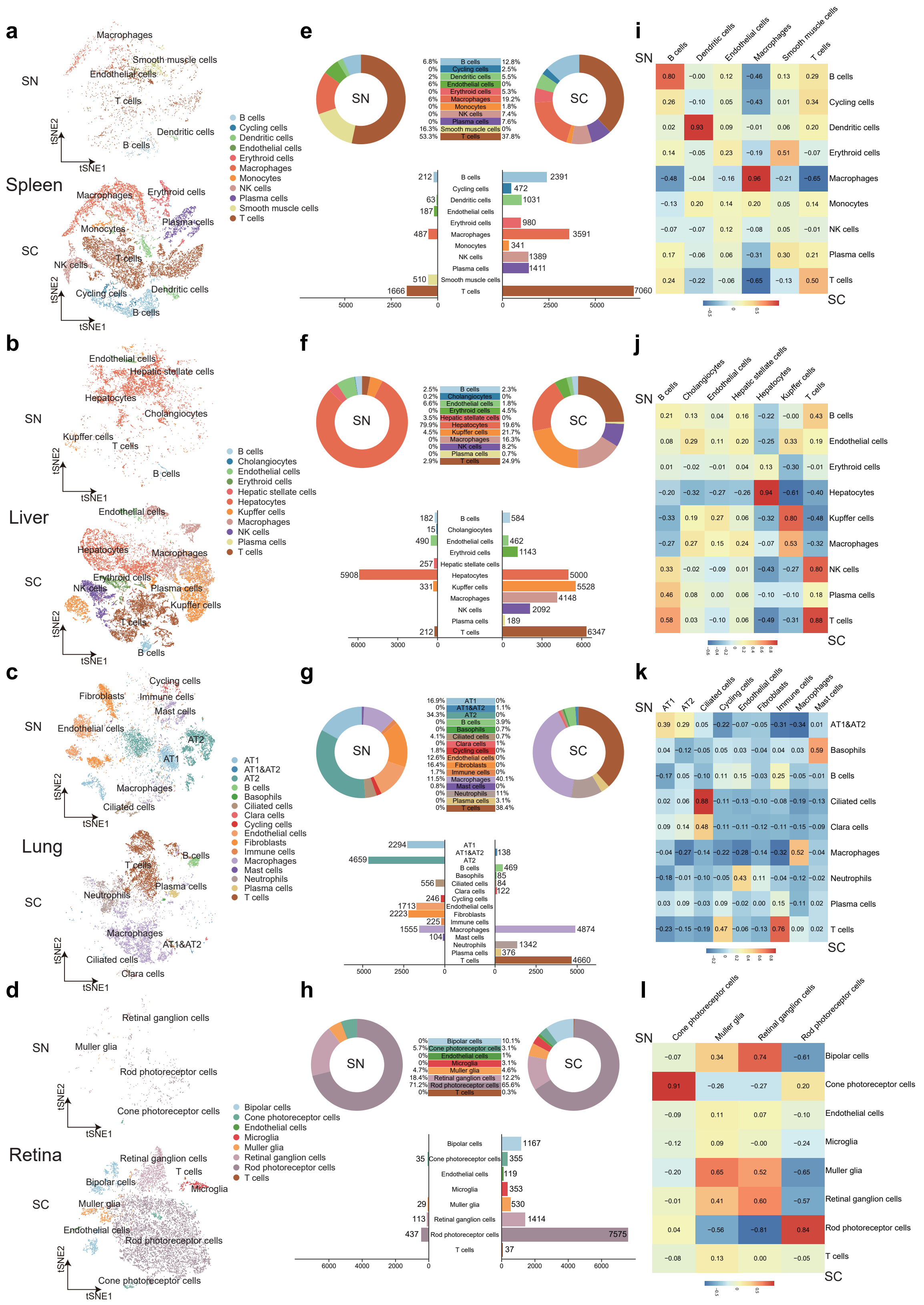

Figure S1. Comparative analysis of scRNA-seq and snRNA-seq datasets from the spleen, liver, lung, and retina.

a-d. t-SNE visualization of single-cell transcriptome from the spleen (a), liver (b), lung (c), and retina (d). Cells are color-coded according to the batches and types of libraries (top) and according to the cell types (bottom). SN, snRNA-seq; SC, scRNA-seq. **e-h.** Comparison of cell type detection and composition in the spleen (e), liver (f), lung (g), and retina (h) between scRNA-seq and snRNA-seq. i-l. Correlation analysis based on the expression of highly variable genes (HVGs) between cell types in the spleen (i), liver (j), lung (k), and retina (l) from scRNA-seq and snRNA-seq.

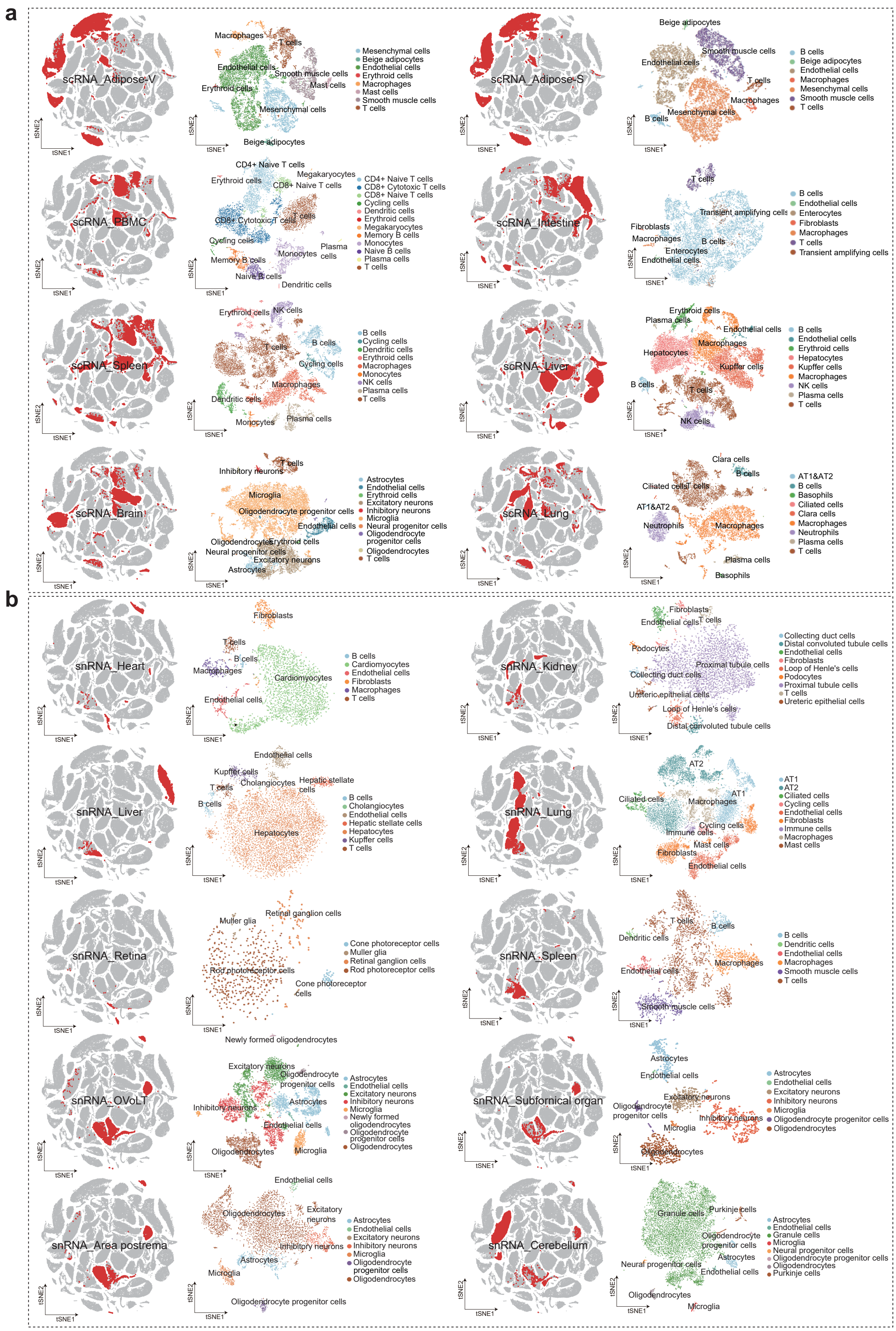

Figure S2. Visualization and annotation of the 58 major cell types

Characterization of different cell types captured by scRNA (**a**) and snRNA (**b**) sequencing of Visceral adipose (scRNA-seq), Subcutaneous adipose (scRNA-seq), PBMC (scRNA-seq), Intestine (scRNA-seq), Spleen (scRNA-seq), Liver (scRNA-seq), Brain (scRNA-seq), Lung (scRNA-seq), Heart (snRNA -seq), Kidney (snRNA-seq), Liver (snRNA-seq), Lung (snRNA-seq), Retina (snRNA-seq), Spleen (snRNA-seq), OVoLT, vascular organ of lamina terminalis (snRNA-seq), Subfomical organ (snRNA-seq), Area postrema (snRNA-seq), and Cerebellum (snRNA-seq). Also see extended Supplementary Data S3 and S4.

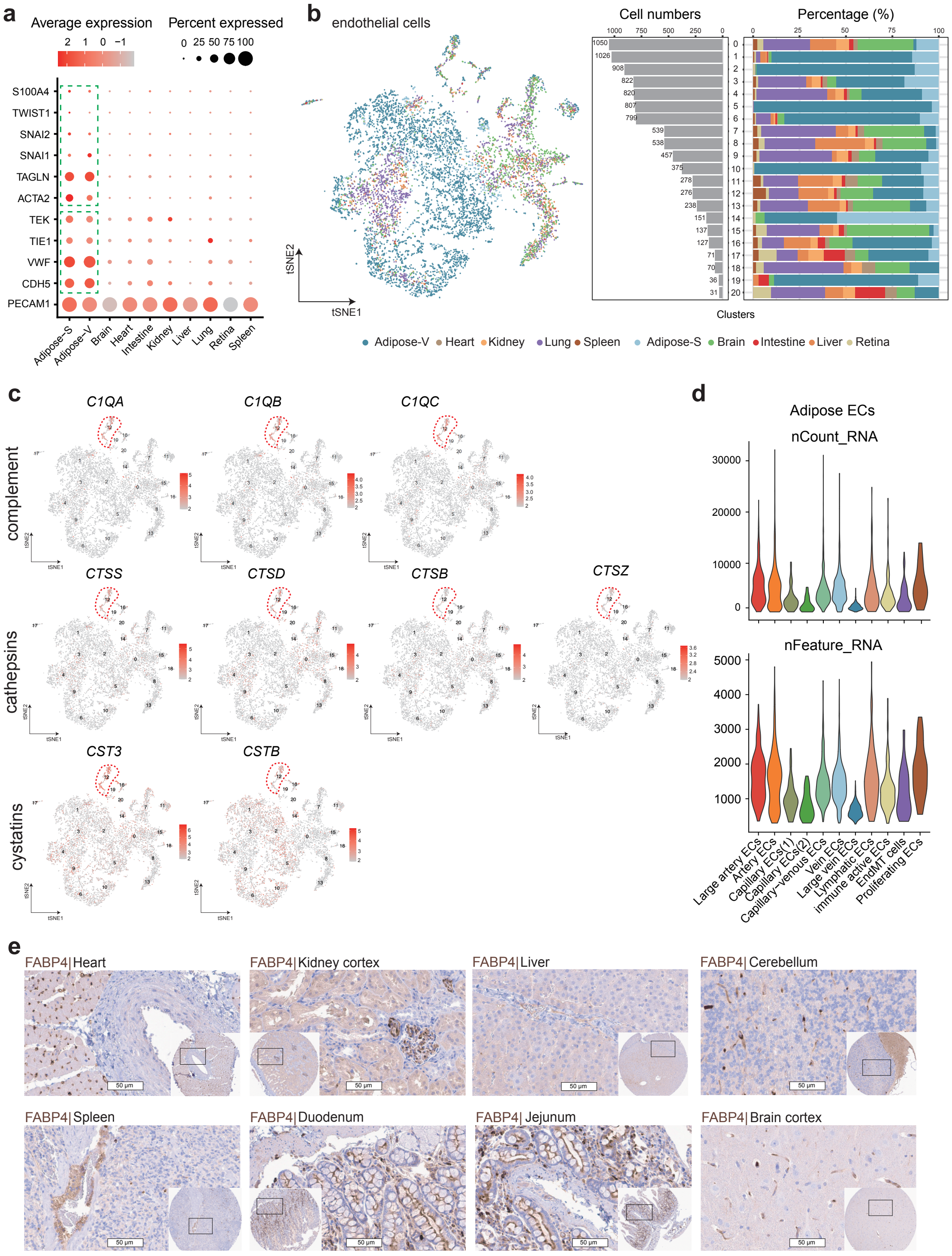

Figure S3. EndMT cells in the adipose tissues and FABP4+ ECs across tissues

a. Dot-plot of EC and mesenchymal cell marker genes across tissues, highlighted in boxes. **b**. t-SNE visualization of ECs according to tissue types (left), bar plot of total number of ECs in each cluster (middle), and the fraction of each cluster of ECs in each tissue (right). **c**. Feature-plots of complement, cathepsins, and cystatins genes in the immune active EC cluster (c12). **d**. Violin-plot showing the nFeature_RNA and nCount_RNA in adipose ECs subtypes. **e**. IHC validation of capillary EC specific marker FABP4 in eight different pig tissues. Box, enlarged region of focus.

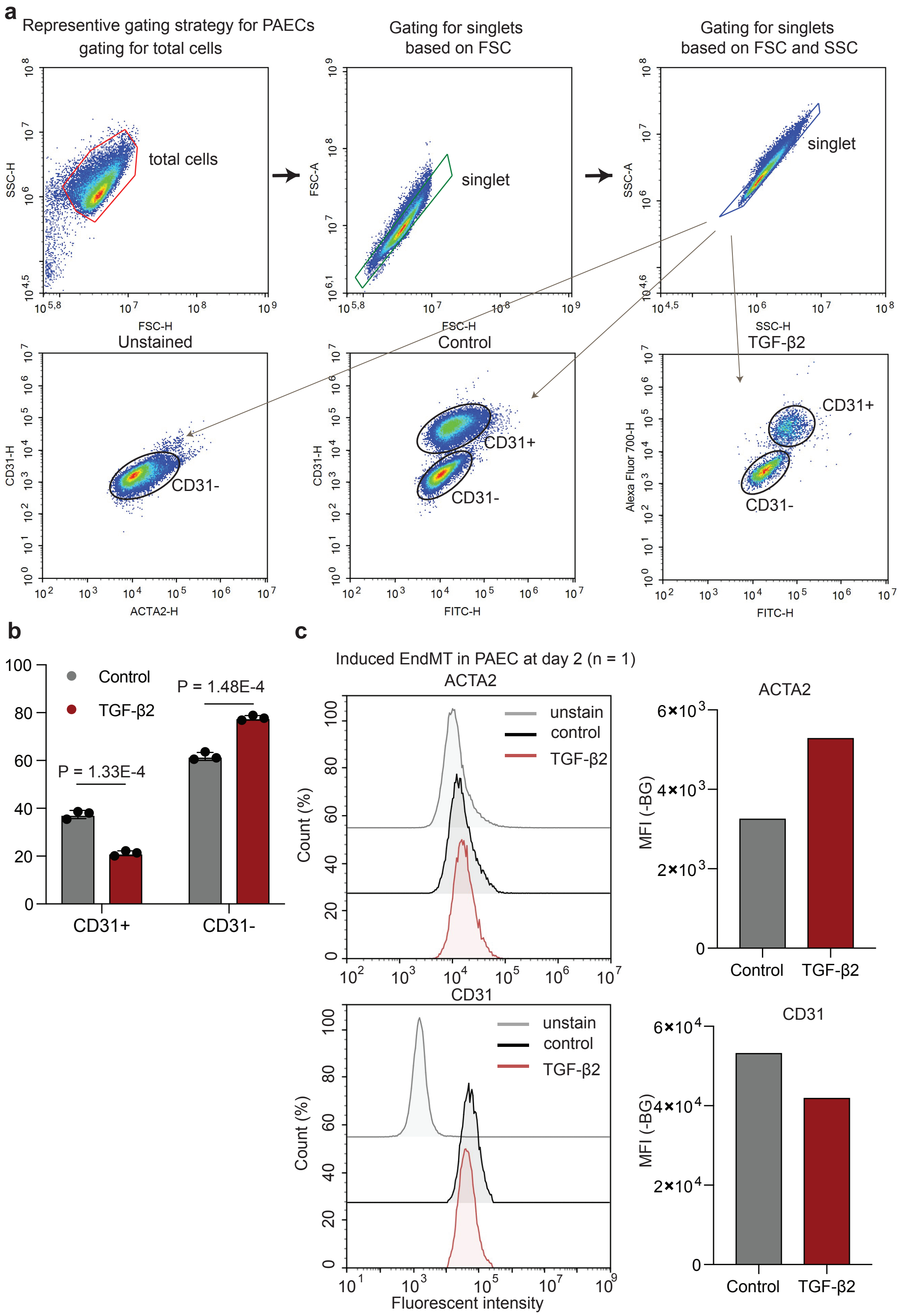

Figure S4. Induced EndMT cells after TGFb2 treatment.
a. Representative gating strategy for ECs analysis. **b.** Fractions of CD31+ and CD31- in the cultured PAECs after 5 days treatment and in controls (n=3, p values are from two-sided t-test). Values are presented as mean SD. **c**. Induced EndMT in PAEC at day 2 (n=1). MFI, median fluorescence intensity. BG, background MFI signal of unstain.

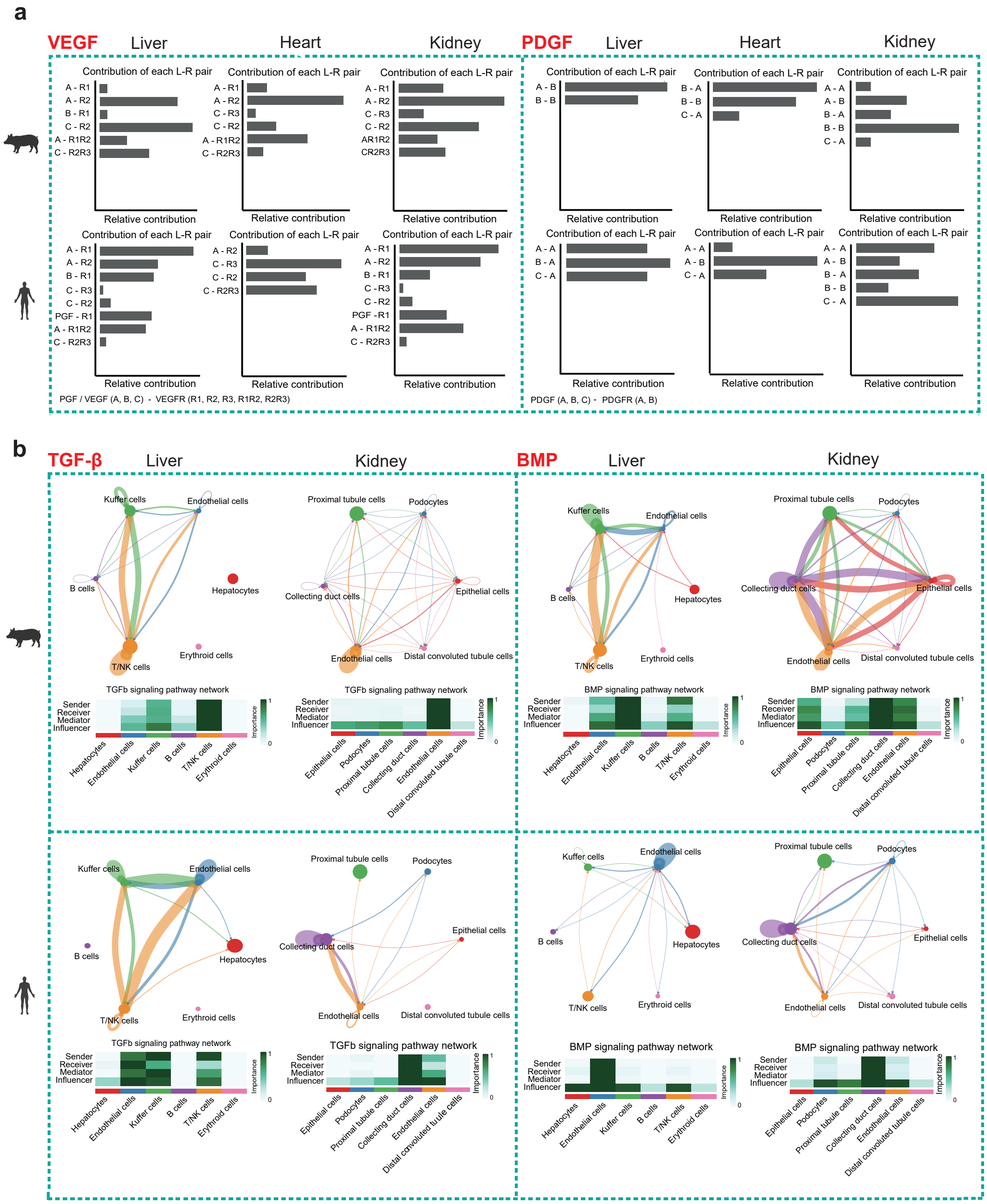

Figure S5. Comparison of cell communication and signaling pathways between pig and human.

a. The relative contribution of each ligand-receptor pair to the overall VEGF and PDGF signaling pathway in liver, heart, and kidney. **b.** Comparison of cell-cell communication of TGF-β and BMP signaling pathway in liver and kidney. Extended data for Figure 5. Pig and human icons are created with BioRender.com.

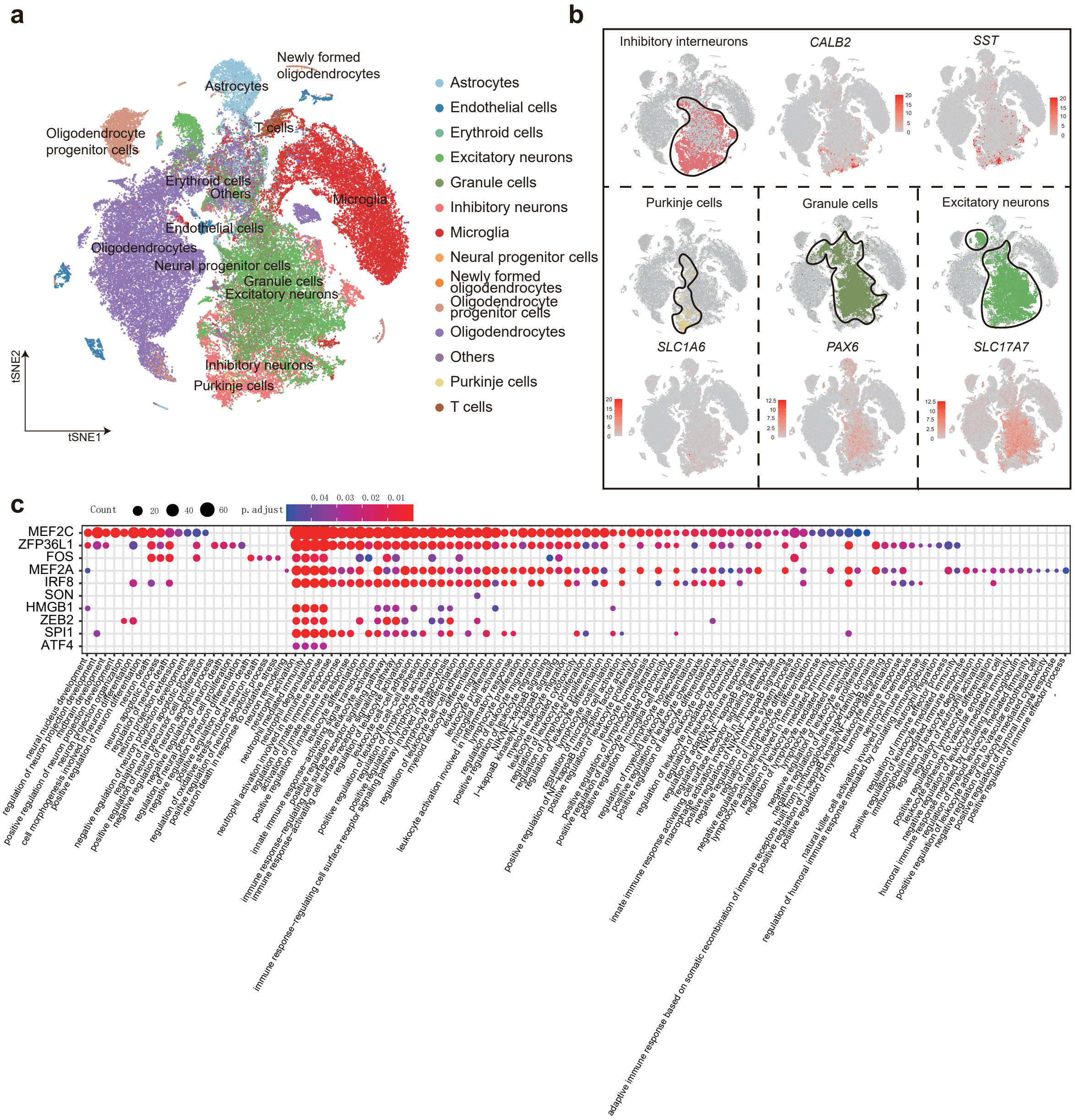

Figure S6. Cell types in brain regions and GO analysis of microglia.
a. t-SNE visualization of cell types in different brain regions. **b**. Feature-plots of gene expression in excitatory neurons (SLC17A7), granule cells (PAX6), inhibitory neurons (CALB2, SST), and Purkinje cells (SLC1A6). **c**. GO enrichment related to cellular functions of predicted target genes of top 10 conversed TFs in microglia.
