# Supplementary material for: Endothelial cell heterogeneity and microglia regulons revealed by a pig cell landscape at single-cell level
Source: Nat Commun. 2022 Jun 24;13:3620. doi: 10.1038/s41467-022-31388-z (PMC9232580; doi:10.1038/s41467-022-31388-z)
Supplement: Supplementary file 3 — Descriptions of additional supplementary data [file 41467_2022_31388_MOESM3_ESM.pdf]

Descriptions of additional supplementary data files

Supplementary Data 1. Metadata of all samples, sequencing, sequencing quality control and cells.

Supplementary Data 2. Comparison analysis of scRNA-seq and snRNAseq.

Supplementary Data 3. Summary of clusters, corresponding cell types, and cell type specific markers used for annotation in all 20 tissues.

Supplementary Data 4. Top 50 markers for each cell cluster.

Supplementary Data 5. List of enriched GOs based on marker genes for retina cells.

Supplementary Data 6. List of enriched GOs based on marker genes for kidney cells.

Supplementary Data 7. List of numbers and fractions of ECs from all tissues.

Supplementary Data 8. List of DEGs and GOs in the 21 EC clusters.

Supplementary Data 9. Marker genes for the EC subtypes in adipose tissues.

Supplementary Data 10. List of the liver, kidney, and heart datasets from pigs and humans.

Supplementary Data 11. List of the cell numbers of single cell transcriptome in the liver, kidney, and heart from pigs and humans.

Supplementary Data 12. Sample information of 13 different species.

Supplementary Data 13. List of conserved TF-target pairs in 13 different species.

Supplementary Data 14. List of antibodies used in this study.
