# Supplementary material for: Endothelial cell heterogeneity and microglia regulons revealed by a pig cell landscape at single-cell level
Source: Nat Commun. 2022 Jun 24;13:3620. doi: 10.1038/s41467-022-31388-z (PMC9232580; doi:10.1038/s41467-022-31388-z)
Supplement: Supplementary file 18 — Reporting Summary [file 41467_2022_31388_MOESM18_ESM.pdf]

nature portfolio

Corresponding author(s): Dongsheng Chen, Lin Lin, Yonglun Luo

Last updated by author(s): Jun 2, 2022

Reporting Summary

Nature Portfolio wishes to improve the reproducibility of the work that we publish. This form provides structure for consistency and transparency in reporting. For further information on Nature Portfolio policies, see our Editorial Policies and the Editorial Policy Checklist.

Statistics

For all statistical analyses, confirm that the following items are present in the figure legend, table legend, main text, or Methods section.

n/a	Confirmed	
☐	☒	The exact sample size (*n*) for each experimental group/condition, given as a discrete number and unit of measurement
☐	☒	A statement on whether measurements were taken from distinct samples or whether the same sample was measured repeatedly
☐	☒	The statistical test(s) used AND whether they are one- or two-sided<br>*Only common tests should be described solely by name; describe more complex techniques in the Methods section.*
☒	☐	A description of all covariates tested
☐	☒	A description of any assumptions or corrections, such as tests of normality and adjustment for multiple comparisons
☐	☒	A full description of the statistical parameters including central tendency (e.g. means) or other basic estimates (e.g. regression coefficient) AND variation (e.g. standard deviation) or associated estimates of uncertainty (e.g. confidence intervals)
☐	☒	For null hypothesis testing, the test statistic (e.g. *F*, *t*, *r*) with confidence intervals, effect sizes, degrees of freedom and *P* value noted<br>*Give P values as exact values whenever suitable.*
☒	☐	For Bayesian analysis, information on the choice of priors and Markov chain Monte Carlo settings
☒	☐	For hierarchical and complex designs, identification of the appropriate level for tests and full reporting of outcomes
☐	☒	Estimates of effect sizes (e.g. Cohen's *d*, Pearson's *r*), indicating how they were calculated

Our web collection on statistics for biologists contains articles on many of the points above.

Software and code

Policy information about availability of computer code

Data collection	Libraries for scRNA-seq were generated using the GemCode Single Cell 3' Gel Bead and Library kit (v3 Chemistry) from 10x Genomics. After library construction, the library conversion was performed using the MGIEasy Universal DNA Library Preparation reagent kit (BGI, Shenzhen, China) for compatibility. For snRNA-seq, The mRNA capture was operated on a DNBelab C4 device (MGI). cDNA amplification and libraries construction were generated using the MGI DNBelab C series reagent kit (MGI) following the manufacturer's instructions. All the libraries were sequenced on the DNBSEQ-T7 platform.
Data analysis	Cell Ranger 3.0.2 (10x Genomics) was used to process the raw sequencing data. The reference genome was downloaded from the Ensemble assembly: Sscrofa11.1. After filtering, unsupervised clustering was performed using Seurat v3 (v3.2.2). Gene Ontology (GO) analysis was using in the clusterProfiler package (v3.12.0). The Pearson correlation coefficients of cell types were calculated using the average expression of top 3000 highly variable features and visualized using pheatmap R package (v1.0.12). Intercellular communication analysis was conducted using CellChat (v0.0.1) R package with default parameters. TFs gene list was downloaded from the animalTFDB 3.0. Only genes expressed in more than 5% of corresponding cell types were subjected to GENIE3 (v1.8.0) to infer putative regulatory circuits from expression data using tree-based ensemble methods. Conserved regulomes were visualized using the igraph (v1.2.6) R package.

For manuscripts utilizing custom algorithms or software that are central to the research but not yet described in published literature, software must be made available to editors and reviewers. We strongly encourage code deposition in a community repository (e.g. GitHub). See the Nature Portfolio guidelines for submitting code & software for further information.

Data

Policy information about availability of data

All manuscripts must include a data availability statement. This statement should provide the following information, where applicable:

- Accession codes, unique identifiers, or web links for publicly available datasets
- A description of any restrictions on data availability
- For clinical datasets or third party data, please ensure that the statement adheres to our policy

The data generated in this study have been deposited in the CNSA of CNGBdb database under accession code CNP0002165 [https://db.cngb.org/search/project/CNP0002165/]. The data generated in this study have also been deposited in the gene expression omnibus database under accession code GSE196055 [https://www.ncbi.nlm.nih.gov/geo/query/acc.cgi?acc=GSE196055] and GSE193975 [https://www.ncbi.nlm.nih.gov/geo/query/acc.cgi]. All matrix data can be downloaded from the PCA database [https://dreamapp.biomed.au.dk/pigatlas/]. Source data are provided with this paper. /). All other relevant data supporting the key findings of this study are available within the article and its Supplementary Information files or from the corresponding author upon reasonable request.

Field-specific reporting

Please select the one below that is the best fit for your research. If you are not sure, read the appropriate sections before making your selection.

[x] Life sciences [ ] Behavioural & social sciences [ ] Ecological, evolutionary & environmental sciences

For a reference copy of the document with all sections, see nature.com/documents/nr-reporting-summary-flat.pdf

Life sciences study design

All studies must disclose on these points even when the disclosure is negative.

Sample size	In total, we generated the datasets from 20 different pig organs/tissues, yielding a total of 222,526 cells after quality control.
Data exclusions	For ScRNA-Seq data,We excluded low quality cells through quality control pipeline. In Figure 5, S5, the celltypes that are specific existing in pig or human kidney, heart, and liver were excluded. In Figure 6, S6, we only used microglia for further analysis, the other cell types were excluded.
Replication	Some canonical markers for celltypes and findings derived from scRNA-seq analysis were confirmed by Immunohistochemical (IHC) staining (Figure 2e, 2g, 2k, 3e, 6c) and iimmunofluorescence (IF) staining (Figure 2i, 2j). Representative IHC and IF images (n = 3, individual pigs) were provided in the figure and supplementary figures. Results in Figure 4g, 4h, and Supplementary Figure S4b were performed in three experimental replicates.
Randomization	No randomization was applied for the study.
Blinding	For scRNA/snRNA-seq data analysis, due to no treatment was done, there was no need for blinding procedure. For cell experiments, blinding was not possible because individual groups of cells received different treatments. IHC staining results were assessed by two independent pathologists who were blinded to group allocation during data analysis.

Reporting for specific materials, systems and methods

We require information from authors about some types of materials, experimental systems and methods used in many studies. Here, indicate whether each material, system or method listed is relevant to your study. If you are not sure if a list item applies to your research, read the appropriate section before selecting a response.

Materials & experimental systems

n/a	Involved in the study	
[ ]	[x]	Antibodies
[x]	[ ]	Eukaryotic cell lines
[x]	[ ]	Palaeontology and archaeology
[ ]	[x]	Animals and other organisms
[x]	[ ]	Human research participants
[x]	[ ]	Clinical data
[x]	[ ]	Dual use research of concern

Methods

n/a	Involved in the study	
[x]	[ ]	ChIP-seq
[x]	[ ]	Flow cytometry
[x]	[ ]	MRI-based neuroimaging

Antibodies

Antibodies used

Antibody Species Source Catalog number Dilution Figure
Rabbit anti-PECAM1 human, pig HPA Cat# HPA004690 1:50 Fig.3j

Mouse anti-VWF human, pig Atlas Antibodies Cat# CL1957 1:7000 Fig.3i
Mouse anti-ACTA2 human, pig Atlas Antibodies Cat# CAB013531 1:100 Fig.3j
Rabbit anti-TAGLN human, pig HPA Cat# HPA019467 1:100 Fig.3i
Rabbit Anti-human RHO human, pig HPA Cat# HPA013440 1:150 Fig.2e
Rabbit Anti-human ARR3 human, pig HPA Cat# HPA063129 1:2400 Fig.2e
Rabbit Anti-human GNG13 human, pig HPA Cat# HPA046272 1:900 Fig.2e
Rabbit Anti-human CRX human, pig HPA Cat# HPA036762 1:1200 Fig.2e
Rabbit Anti-human CDHR1 human, pig HPA Cat# HPA036819 1:900 Fig.2e
Rabbit Anti-human RBP3 human, pig HPA Cat# HPA041301 1:1000 Fig.2j
Rabbit Anti-human NPHS2 human, pig HPA Cat# HPA049486 1:600 Fig.2j
Rabbit Anti-human SLC12A1 human, pig HPA Cat# HPA014967 1:250 Fig.2j
Rabbit Anti-human GATA2 human, pig HPA Cat# HPA005633 1:60 Fig.2j
Rabbit Anti-human AQP2 human, pig HPA Cat# HPA046834 1:600 Fig.2j
Rabbit Anti-human PAX2 human, pig HPA Cat# HPA047704 1:1000 Fig.2j
Rabbit Anti-human FABP4 human, pig HPA Cat# HPA002188 1:200 Fig.3k; Fig. S3e
Rabbit Anti-human SLC1A6 human, pig HPA Cat# HPA041505 1:2500 Fig. 6c
Rabbit Anti-human CALB2 human, pig HPA Cat# HPA007305 1:6000 Fig. 6c
Rabbit Anti-human PAX6 human, pig HPA Cat# HPA030775 1:20 Fig. 6c
Rabbit Anti-human SLC17A7 human, pig HPA Cat# HPA063679 1:1400 Fig. 6c
Rabbit Anti-human SST human, pig HPA Cat# HPA019472 1:1500 Fig. 6c
Alexa Fluor® 488 Mouse Anti-ACTA2 human, pig R&D Cat# IC1420G 1:100 Fig. 4g,h; Fig. S4
Alexa Fluor® 700 Rat Anti-pig CD31 pig R&D Cat# FAB33871N 1:100 Fig. 4g; Fig. S4
BV421 Mouse Anti-CD45 human, pig BD Bioscience Cat# 563879 1:100 Fig. S4
FITC Mouse Anti-human CD31 human BD Bioscience Cat# 555445 1:100 Fig. 4h; Fig. S4

Validation

All antibodies used are commercially available and their manufacturers provided their validation documents. They were validated for IF and/or IHC staining.
1. Rabbit anti-pig PECAM1, HPA, Cat# HPA004690
https://www.atlasantibodies.com/products/antibodies/primary-antibodies/triple-a-polyclonals/pecam1-antibody-hpa004690/
2. Mouse anti-pig VWF, Atlas Antibodies, Cat# CAB072875
https://www.proteinatlas.org/ENSG00000110799-VWF/antibody
3. Mouse anti-pig ACTA2, Atlas Antibodies, Cat# CAB013531
https://www.proteinatlas.org/ENSG00000107796-ACTA2/antibody
4. Rabbit anti-pig TAGLN, HPA, Cat# HPA019467
https://www.atlasantibodies.com/products/antibodies/primary-antibodies/triple-a-polyclonals/tagln-antibody-hpa019467/
5. Anti-pig RHO, HPA, Cat# HPA013440
https://www.atlasantibodies.com/products/antibodies/primary-antibodies/triple-a-polyclonals/rho-antibody-hpa013440/
6. Anti-pig ARR3, HPA, Cat# HPA063129
https://www.atlasantibodies.com/products/antibodies/primary-antibodies/triple-a-polyclonals/arr3-antibody-hpa063129/
7. Anti-pig GNG13, HPA, Cat# HPA046272
https://www.atlasantibodies.com/products/antibodies/primary-antibodies/triple-a-polyclonals/gng13-antibody-hpa046272/
8. Anti-pig CRX, HPA, Cat# HPA036762
https://www.atlasantibodies.com/products/antibodies/primary-antibodies/triple-a-polyclonals/crx-antibody-hpa036762/
9. Anti-pig CDHR1, HPA, Cat# HPA036819
https://www.atlasantibodies.com/products/antibodies/primary-antibodies/triple-a-polyclonals/cdhr1-antibody-hpa036819/
10. Anti-pig RBP3, HPA, Cat# HPA041301
https://www.atlasantibodies.com/products/antibodies/primary-antibodies/triple-a-polyclonals/rbp3-antibody-hpa041301/
11. Anti-pig NPHS2, HPA, Cat# HPA049486
https://www.atlasantibodies.com/products/antibodies/primary-antibodies/triple-a-polyclonals/nphs2-antibody-hpa049486/
12. Anti-pig SLC12A1, HPA, Cat# HPA014967
https://www.sigmaaldrich.com/DK/en/product/sigma/hpa014967
13. Anti-pig GATA2, HPA, Cat# HPA005633
https://www.atlasantibodies.com/products/antibodies/primary-antibodies/triple-a-polyclonals/gata2-antibody-hpa005633/
14. Anti-pig AQP2, HPA, Cat# HPA046834
https://www.atlasantibodies.com/products/antibodies/primary-antibodies/triple-a-polyclonals/aqp2-antibody-hpa046834/
15. Anti-pig PAX2, HPA, Cat# HPA047704
https://www.atlasantibodies.com/products/antibodies/primary-antibodies/triple-a-polyclonals/pax2-antibody-hpa047704/
16. Anti-pig FABP4, HPA, Cat# HPA002188
https://www.atlasantibodies.com/products/antibodies/primary-antibodies/triple-a-polyclonals/fabp4-antibody-hpa002188/
17. Anti-pig SLC1A6, HPA, Cat# HPA041505
https://www.atlasantibodies.com/products/antibodies/primary-antibodies/triple-a-polyclonals/slc1a6-antibody-hpa041505/
18. Anti-pig SALB2, HPA, Cat# HPA007305
https://www.atlasantibodies.com/products/antibodies/primary-antibodies/triple-a-polyclonals/calb2-antibody-hpa007305/
19. Anti-pig PAX6, HPA, Cat# HPA030775
https://www.atlasantibodies.com/products/antibodies/primary-antibodies/triple-a-polyclonals/pax6-antibody-hpa030775/
20. Anti-pig SLC17A7, HPA, Cat# HPA063679
https://www.atlasantibodies.com/products/antibodies/primary-antibodies/triple-a-polyclonals/slc17a7-antibody-hpa063679/
21. Anti-pig SST, HPA, Cat# HPA019472
https://www.atlasantibodies.com/products/antibodies/primary-antibodies/triple-a-polyclonals/sst-antibody-hpa019472/

22. Human alpha-smooth muscle actin, R&D, Cat# IC1420G
https://www.bio-techne.com/p/antibodies/human-alpha-smooth-muscle-actin-alexa-fluor-488-conjugated-antibody-1a4_ic1420g
23. Porcine CD31/PECAM1, R&D, Cat# FAB33871N
https://www.bio-techne.com/p/antibodies/porcine-cd31-pecam-1-alexa-fluor-700-conjugated-antibody-377537_fab33871n
24. Mouse anti-human CD45, BD Bioscience, Cat# 563879
https://www.bdbiosciences.com/en-dk/products/reagents/flow-cytometry-reagents/research-reagents/single-color-antibodies-ruo/bv421-mouse-anti-human-cd45.563880
25. Mouse anti-human CD31, BD Bioscience, Cat# 555445
https://www.bdbiosciences.com/en-dk/products/reagents/flow-cytometry-reagents/research-reagents/single-color-antibodies-ruo/fitc-mouse-anti-human-cd31.560984

Animals and other organisms

Policy information about studies involving animals; ARRIVE guidelines recommended for reporting animal research

Laboratory animals	The samples of pig organs were collected from a local slaughterhouse. We did not adjust our analysis for age and gender. Details samples processing steps are provided in the materials and methods. Species, strain, gender and age of pigs are listed in supplementary data 1.
Wild animals	Single cell RNA sequencing data for wild animals in this study were collected from the online published database.
Field-collected samples	No field-collected samples were used.
Ethics oversight	The study was approved by the Institutional Review Board on the Ethics Committee of BGI (Approval letter reference number BGI-NO.BGI-IRB18135-T1). All experimental procedures were conducted following the guidelines of the experimental animals. All the applicable institutional and national guidelines for the care and welfare of animals were followed.

Note that full information on the approval of the study protocol must also be provided in the manuscript.
